# Malignant Arrhythmia and Cardiac Arrest Following Intentional Yew Tree Leaf Ingestion Salvaged by VA‐ECMO

**DOI:** 10.1002/ccr3.70243

**Published:** 2025-03-13

**Authors:** William Ries, Muhammad Faisal, Thomas Kirk, Aqib Hafeez, Laura Vincent, David Clarke, Graham Barker

**Affiliations:** ^1^ General Medicine, John Radcliffe Hospital Oxford University Hospitals NHS Foundation Trust Oxford UK; ^2^ Emergency Department, John Radcliffe Hospital Oxford University Hospitals NHS Foundation Trust Oxford UK; ^3^ Oxford Critical Care, John Radcliffe Hospital Oxford University Hospitals NHS Foundation Trust Oxford UK

**Keywords:** extracorporeal membrane oxygenation, plant poisoning, resuscitation, toxicology

## Abstract

This case emphasizes the role of multidisciplinary involvement and early decision‐making in yew tree (*Taxus baccata)* poisoning. *Taxus baccata* contains taxine alkaloids that predispose to malignant arrhythmia. Here, we present a case of *Taxus baccata* toxicity presenting with refractory cardiac arrest, salvaged ultimately by VA‐ECMO.

**Summary**

- Expedited institution of VA‐ECMO may maximize chances of salvaging *Taxus baccata* toxicity.
- Combination therapy of intravenous sodium bicarbonate, digoxin‐specific antibody, lipid emulsion therapy, hydrocortisone, and gastric decontamination may serve as a therapeutic bridge until VA‐ECMO can be established.
- More broadly, complete biochemical and neurological recovery is possible following recurrent asystolic and PEA arrest with aggressive supportive care.

## 1 Introduction

*Taxus baccata* has been known for its noxious effects since ancient times [1]. There is no known antidote [2]. Treatment strategies are largely informed by case reports of successful therapies internationally [3, 4]. Early institution of venoarterial extracorporeal membrane oxygenation (VA‐ECMO) is evolving as a central pillar of successful treatment of clinically significant overdose [5]. Our case adds to the literature for two reasons. First, it details the use of several, not just one, temporizing treatment strategy all in one patient. These strategies could serve as a ‘treatment bundle’ whilst VA‐ECMO services are contacted. Second, it adds to the expanding evidence base that early institution of VA‐ECMO may give patients the best chance of achieving a full biochemical and neurological recovery from taxine poisoning.

## 2 Case History

A woman in her 20s ingested approximately 30 g (equivalent to a dose of 0.46 g of leaves per kg) of *Taxus baccata* in a concerted suicide attempt. She had no notable past medical history but had recently been an inpatient at a psychiatric hospital for suicidal ideation on a background of informally diagnosed emotionally unstable personality disorder. After ingesting the leaves, she called her father, informing him of her actions. The father collected his daughter and drove her to the local hospital in a private vehicle. They arrived at the local emergency department 90 min after ingestion, but the patient collapsed in the hospital grounds before being able to enter the building.

## 3 Methods

**FIGURE 1:**
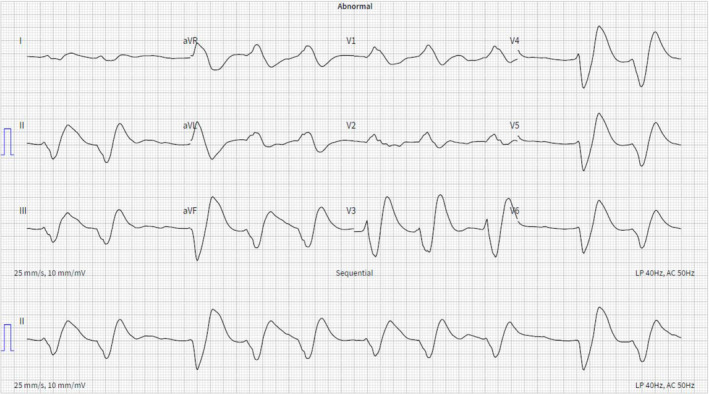
Post‐resuscitation 12 lead‐ECG demonstrating an irregular broad complex rhythm.

Cardiopulmonary resuscitation (CPR) was commenced immediately by a nearby ambulance crew. Cardiac rhythm analysis revealed ventricular fibrillation. The patient was transferred to the emergency department with resuscitation ongoing. Intravenous adrenaline (1 mg of 1 in 10,000 as a bolus) and amiodarone (300 mg as a bolus) were given during the resuscitation attempt in accordance with Advanced Life Support (ALS) UK guidelines [6]. Return of spontaneous circulation (ROSC) was achieved after 17 min. A total of 7 biphasic defibrillations at 200 J were administered during the resuscitation effort, with cardiac rhythms evolving from ventricular fibrillation to ventricular tachycardia to pulseless electrical activity (Table 1). A post‐resuscitation 12‐lead electrocardiogram (ECG) demonstrated an irregular broad complex rhythm (Figure 1). Arterial blood gases revealed a metabolic acidosis with a lactate of 5.8 mmol/L. Glasgow Coma Scale score was 3.

**TABLE 1:** Cardiac rhythms recorded during the resuscitation attempt in the emergency department before being transferred to intensive care unit.

Time	Rhythm	Therapies
Arrival to emergency department	VF	CPR ongoing
+2 min	VF	Defibrillation
+4 min	VF	Defibrillation
+6 min	VF	Defibrillation
+8 min	Pulseless VT	Defibrillation
+9 min	—	Intubated
+10 min	PEA	CPR
+13 min	PEA	CPR
+15 min	PEA	CPR
+17 min	ROSC	ABCDE, central line insertion

The patient underwent tracheal intubation and was ventilated in the emergency department. She remained hypotensive and suffered alternating bouts of wide complex tachy‐ and bradyarrhythmias, with the latter transiently responsive to an intravenous isoprenaline infusion. An intravenous adrenaline infusion was commenced due to extreme hemodynamic instability. The patient became increasingly bradycardic and suffered a second cardiac arrest, with cardiac rhythm analysis demonstrating asystole at 74 min following the initial cardiac arrest. ALS UK guidelines were followed, with CPR and continuous ventilation. An additional 1 mg intravenous adrenaline was given, with ROSC achieved after 1 min of CPR.

Since the patient's father relayed that the patient had ingested yew tree leaves, taxine poisoning was felt to be the most likely etiology for the patient's recurrent cardiopulmonary arrests. An emergency oesophagogastroduodenoscopy (OGD) was performed at the bedside. Several tree leaves were seen in the gastric body, which were successfully retrieved using a Roth net and aspiration. The gastric mucosa was then washed copiously and aspirated completely.

In attempts to mitigate the destabilizing arrhythmogenic effects of *Taxus baccata*, the patient received two 80 mg doses of intravenous digoxin‐specific antibody (Digibind), 100 mL of intravenous 8.4% sodium bicarbonate, 4 mmol of intravenous magnesium sulfate, and an intravenous amiodarone infusion [7]. Intravenous lipid emulsion therapy was also administered, first as a 100 mL bolus and then as an infusion [8]. 200 mg of intravenous hydrocortisone was administered due to previous case reports indicating benefit when used in Yew tree overdose [9].

Despite these measures, the patient continued to suffer progressive hemodynamic instability and was thus referred to the regional ECMO service. She was established on peripheral VA‐ECMO approximately 7 h after *Taxus baccata* ingestion. VA‐ECMO seeks to provide transient mechanical circulatory support by means of an external pump and oxygenator [10]. For its peripheral use, the native circulation is connected to the pump and oxygenator via an inflow venous cannula typically from the femoral vein, with outflow/return connected to the femoral artery [10].

Computed tomography (CT) imaging of the head on the day of presentation revealed preserved gray‐white matter differentiation, no acute vascular territory cortical infarction or hemorrhage, no midline shift, or intra‐ or extra‐axial collections. The ventricles, subarachnoid spaces, and cerebellar hemispheres were normal. CT imaging of the chest, abdomen, and pelvis was unremarkable, with the exception of there being small bilateral pleural effusions with associated bibasal atelectasis.

## 4 Results

The patient was successfully decannulated from VA‐ECMO on day 3 of tertiary center admission. Decannulation was complicated by a right femoral artery embolus for which she underwent successful embolectomy. After transfer back to the referring center, the patient was weaned from sedation and ventilatory requirements without incident. The patient was soon afterwards discharged from the hospital with close psychiatric support in the community. Her liver and renal function at discharge were within normal parameters (serum creatinine 48 umol/L, ALT 35 IU/L, and ALP 76 IU/L). She achieved full neurological recovery before discharge with no persisting focal deficits.

A bedside transthoracic echocardiogram (TTE) immediately following her cardiac arrest showed evidence of right ventricular volume and pressure overload, which is common following cardiopulmonary arrest. A subsequent TTE and transoesophageal echocardiogram (TOE) on Day 2 of VA‐ECMO demonstrated normal right ventricular size and function. A cardiac magnetic resonance scan performed 2 months following discharge confirmed sustained normal cardiac function with no evidence of cardiomyopathy.

## 5 Discussion

*Taxus baccata* has long been known for its lethal side effects [1]. Folklore reports that Boudica, the Celtic queen of ancient Britain, attempted suicide by ingesting the tree's evergreen leaves, distraught by her defeat to the Romans [11]. In modern times, the tree has gained traction amongst patients with strong suicidal intent as a readily available toxic substance, which in overdose has no known antidote [12]. Its lethality derives from the alkaloid toxin taxine B, which predisposes to malignant arrhythmia by disrupting myocyte voltage‐gated calcium and sodium channels [1, 11, 12]. The malignant electrophysiological traces seen in this case demonstrate the effect of extreme membrane channel blockade on cardiac rhythm—agonal sinusoidal rhythms alternating with recurrent bouts of ventricular tachycardia followed by junctional escape rhythms.

A literature search reveals several dozen case reports of *Taxus baccata* poisoning over the past two decades. Recognized treatment options include intravenous sodium bicarbonate to correct profound metabolic acidosis and encourage intracellular sodium transport [8]; early gastric decontamination either by emergency endoscopy or activated charcoal; administration of intravenous Digibind, as digoxin‐specific Fab fragments have been found to bind taxines due to their structural similarity to digoxin [7]; and administration of lipid emulsion therapy, as taxine B is believed to be lipophilic [8].

In recent decades, the anti‐mitotic effects of *Taxus baccata* have been harnessed as chemotherapy agents—giving rise to the taxane class of cancer treatments, including medications such as paclitaxel and docetaxel [13]. Strategies for mitigating taxane‐class toxicity can also be trialed in intentional *Taxus baccata* poisoning [9]. For example, in this case, we also used intravenous hydrocortisone.

Other case reports have shown transient stabilizing effects of varying interventions—for example, Farag et al. demonstrating conversion of asystole to a broad complex tachycardia with administration of Digibind [7]. In our patient, we trialed all these treatments in tandem—Digibind, lipid emulsion therapy, intravenous sodium bicarbonate and hydrocortisone, and gastric decontamination. It is difficult to identify a direct benefit from any one single intervention we made. Instead, our case indicates that these treatments may form part of a best supportive care bundle that can act as a bridge until VA‐ECMO can be commenced [3, 8]. It also serves as a reminder that an excellent outcome is possible in what may seem like an unsalvageable presenting clinical state, both biochemically and hemodynamically.

This case highlights that complete neurological and cardiovascular recovery is possible with extensive rapid multi‐specialty input and early institution of VA‐ECMO. It also supports the use of several medications as potential temporizing measures until VA‐ECMO can be established.

## Author Contributions

**William Ries:** writing – original draft, writing – review and editing. **Muhammad Faisal:** writing – original draft, writing – review and editing. **Thomas Kirk:** writing – original draft, writing – review and editing. **Aqib Hafeez:** resources, supervision. **Laura Vincent:** supervision, writing – review and editing. **David Clarke:** supervision, writing – review and editing. **Graham Barker:** supervision, writing – review and editing.

## Ethics Statement

The authors have nothing to report.

## Consent

Written informed consent was obtained from the patient to publish this report in accordance with the journal's patient consent policy. A signed copy for publication of the case alongside accompanying images is held with the corresponding author.

## Conflicts of Interest

The authors declare no conflicts of interest.

## Data Availability

The data that support the findings of this study are available on request from the corresponding author. The data are not publicly available due to privacy or ethical restrictions.
